# Thyroid Disorders and Serum Selenium Levels in a Southwestern Nigerian Population

**DOI:** 10.1155/ije/6915227

**Published:** 2025-09-23

**Authors:** Olufunmilayo Olubusola Adeleye, Olayinka Olabode Ogunleye, Oluwajimi Olanrewaju Sodipo, Ayotunde Oladunni Ale, Ibironke Jadesola Akinola

**Affiliations:** ^1^Department of Medicine, Lagos State University Teaching Hospital, Ikeja, Lagos State, Nigeria; ^2^Department of Medicine and Pharmacology, Lagos State University College of Medicine, Ikeja, Lagos State, Nigeria; ^3^Department of Family Medicine, Lagos State University Teaching Hospital, Ikeja, Lagos State, Nigeria; ^4^Department of Medicine, Olabisi Onabanjo University Teaching Hospital, Sagamu, Ogun State, Nigeria; ^5^Department of Paediatrics, Lagos State University College of Medicine, Ikeja, Lagos State, Nigeria

## Abstract

**Background/Objective:** Thyroid hormones are central to the regulation of energy expenditure and homoeostasis. An important microelement required for the optimal function of the thyroid gland is selenium. The question of whether Nigerian patients with thyroid disorders are deficient in selenium is what this study aims to answer.

**Methods:** This was a comparative cross-sectional study carried out at a tertiary hospital in Southwest Nigeria. Fifty individuals with various thyroid disorders who gave consent were consecutively recruited and compared with one hundred apparently healthy age and sex-matched controls. Blood samples were collected for free thyroxine, free triiodothyronine, thyroid-stimulating hormone and selenium levels. The thyroid hormones were assayed with enzyme-linked immunoassay. Selenium was measured using inductively coupled plasma optical emission spectroscopy. Data were analysed with SPSS Version 26.

**Results:** The mean age of the subjects and control was 40.3 ± 10.6 and 38.0 ± 9.1 years, respectively (*p*=0.157). The subjects and controls were predominantly females, 80% and 72%, respectively. Thyroid disorders were most prevalent in the 41–50 years age category. The male:female ratio was 1:4. The majority of the cases (60%) had biochemical evidence of hyperthyroidism, 28% were euthyroid and 12% were hypothyroid. Selenium levels were significantly lower among the cases than healthy controls (mean selenium level: 24.9 ± 15.7 and 59.0 ± 35.9 μg/L, respectively, *p* < 0.001). With normal selenium levels of 80 μg/L, all the participants were deficient in selenium. There was no significant difference in selenium levels across the spectrum of thyroid disorders although the mean selenium levels were lowest for the hypothyroid subjects.

**Conclusion:** There is significant selenium deficiency in all subjects with thyroid disorders; apparently, healthy Nigerians are generally deficient. Selenium supplementation is recommended for Nigerian patients with thyroid disease and may be required for the general population.

## 1. Introduction

Thyroid disorders rank as the second commonest endocrine disorders seen in the endocrinology clinic after diabetes mellitus [1]. The prevalence of thyroid disorders is rising globally and this increase is multifactorial [2]. There is paucity of population-based prevalence studies which poses a difficulty to ascertaining the prevalence of thyroid disorders in Africa [2]. Central to the physiology and biochemistry of the thyroid gland are micro elements, namely, iodine, iron, copper, selenium, zinc and calcium [3]. However, iodine and selenium are critical to the function of the thyroid gland. A lot is known about iodine and thyroid disease compared with selenium, for instance, there is high incidence of goitre in iodine-deficient areas while the converse is true in iodine-replete areas. On the other hand, selenium deficiency has been reported to contribute to the occurrence and persistence of endemic goitre in areas of Africa where iodine is routinely added to dietary salts [4].

Selenium was discovered in 1817 by Jons Jacob Berzelius, a Swedish chemist, and it exists as six stable isotopes [5]. The geographical distribution of selenium is variable globally and it is only available through dietary sources depending on the soil content in which food crops and animal fodder are grown [6]. Selenium levels in the soil vary according to the geographical location in Nigeria; levels in the south appear to be higher than that reported for the north [7, 8]. Soil selenium has been shown to be deficient in areas that are prone to erosion in Nigeria [7].

In humans, selenium exerts its biological effects through its presence in selenoproteins paramount of which are thioredoxin reductases, glutathione peroxidase and thyroid hormone deiodinases [9]. These effects are concentration dependent with attendant risk of disease if it is deficient or exists in excessive amounts [9].

The thyroid gland has the highest concentration of selenium in the body (0.2–2 μg/g), and it is essential for optimal thyroid gland function, thyroid hormone biosynthesis and metabolism [10]. During the iodination of tyrosyl residues for the production of thyroid hormones, hydrogen peroxide (H_2_O_2_), a free radical, is produced. The accumulation of H_2_O_2_ is prevented by glutathione peroxidase, a selenoprotein whose activity is diminished in states of selenium deficiency [11, 12]. Supplementation with selenium has been shown to be effective against the development of Hashimoto thyroiditis, reduce the levels of thyroid peroxidase autoantibodies, slow the progression of Graves' orbitopathy and improve the quality of life of patients with Graves' disease [13–15]. The European Group on Graves' Orbitopathy (EUGOGO) currently recommends oral selenium supplementation in patients with mildly active Graves' orbitopathy [16]. Furthermore, the deficiency of selenium has been associated with increased risk of thyroid cancer [12]. Although there is a paucity of research into selenium and thyroid disease in Nigeria, Okunade et al. [17] reported that selenium deficiency is associated with adverse pregnancy outcomes among pregnant women with the human immunodeficiency virus (HIV) in Lagos, Southwest Nigeria. There is an observed trend of increasing prevalence of thyroid disease in our locale, which could be attributable to selenium deficiency. Given the key role this microelement plays in optimal thyroid function, this study therefore sets out to answer the hypothesis that selenium levels are low in patients with thyroid disease and to elucidate the association between selenium status and various thyroid disorders presenting to the endocrinology clinic.

## 2. Methodology

This is a comparative cross-sectional one, which was carried out at the Endocrinology Clinic of the Lagos State University Teaching Hospital (LASUTH), Ikeja, Lagos, between February and June 2023. LASUTH is located within Lagos, a city known as the commercial capital of Nigeria, West Africa. The population of Lagos in year 2023 is reported to be 27.4% of the national population [18].

Fifty (50) consecutive adult patients (18 years and above) who had thyroid disease and gave informed consent were enrolled. One hundred (100) age and sex-matched controls were recruited from apparently healthy LASUTH hospital staff. The study included individuals withvarious thyroid disorders specifically categorised into six groups: hyperthyroidism, hypothyroidism, euthyroid goitre, thyroid nodule, subclinical hypothyroidism, and subclinical hyperthyroidism. Exclusion criteria included significant comorbidities(heart failure and cancer) and use of antithyroid medications for over 2 months. Additionally, individuals taking selenium-containing multivitamins were also excluded.

The sample size was calculated with the formula: *N* = *Z*^2^ × PQ/*D*^2^ where *N* was the desired sample size, *Z* is the standard normal deviate (1.96 or 95% confidence interval), *P* is the prevalence of thyroid disease in Southern Nigeria [1], *Q* is 1-*P* and *D* is the degree of accuracy set at 0.05.

The prevalence from a previous study found to be 2.5% [1] was used resulting in a calculated sample size of 37.4 which was rounded to 50. The ratio of cases to controls was set as 1:2. The study was approved by the LASUTH Health Research and Ethics Committee (Reference number LREC/06/10/2136).

Questionnaires were administered by the attending physician to obtain biodata. The history of thyroid disease, duration of symptoms of hyperthyroidism, hypothyroidism and goitre, family history of thyroid disease and type of drinking water were also documented. Ten millilitres of venous blood samples was taken into appropriate bottles for the assay of free triiodothyronine (FT3), free thyroxine (FT4), thyroid-stimulating hormone (TSH) and selenium. This was centrifuged, separated and stored at −50°C until analysed. The analysis for thyroid hormones was done with the Vedas Family Immunodiagnostic Automated Quantitative Enzyme-Linked Fluorescent Immunoassay [19]. Selenium was measured using inductively coupled plasma optical emission spectroscopy (ICP-OES), which is an advanced analytical technique used for the detection of chemical elements in both organic and inorganic fluids using the Agilent ICP-OES system [20].

### 2.1. Statistical Analysis

The data were analysed with SPSS Version 26. Frequency and percentages were employed to summarise the categorical variables, and continuous variables were summarised with means and standard deviation. ANOVA and Student's *t*-test were used to compare the means of continuous variables while the association between categorical variables was conducted with chi-square. The *p* value was set at less than 0.05.

### 2.2. Definition of Terms

1. Goitre was graded as follows: grade 0—no palpable or visible goitre, grade 1—palpable goitre but not visible with the neck in the normal position and grade 2—swelling in the neck that is visible with the neck in a normal position and consistent with an enlarged thyroid gland [21].
2. Normal serum selenium is 80 μg/L [22].
3. The classification of thyroid disorders: Hyperthyroidism is defined by FT4 > 22.0 pmol/L, FT3 > 6.0 pmol/L and TSH < 0.35 µIu/mL [19]. Hypothyroidism is defined by FT3 < 2.9 pmol/L, FT4 < 6.5 pmol/L and TSH > 10 µIu/mL [19]. Euthyroid goitre is an enlargement of the thyroid gland with normal levels of thyroid hormones; subclinical hypo/hyperthyroidism is when the thyroid hormone levels are normal in the setting of elevated or suppressed TSH levels, respectively [23].

The reference range of thyroid hormones was as follows: FT3—2.9–6.0 pmol/L, FT4—6.5–22.0 pmol/L, TSH—0.35–4.94 µIu/mL and AntiTPO < 5.6 μ/mL [19].

## 3. Results

Five people in the control group were discovered to have either deranged thyroid hormonal profile and were therefore excluded from some of the analysis.

The mean age of the subjects and control was 40.3 ± 10.6 and 38.0 ± 9.1 years, respectively (*p*=0.157). There was no statistically significant difference between the two groups indicating that they were well matched. The subjects and controls were predominantly females, 80% and 72%, respectively. Thyroid disorders were most prevalent in the 41–50 years age category. The male:female ratio was 1:4. Twenty-four percent (24%) of the subjects reported a family history of thyroid disease compared with 6% in the control group.  Table 1 depicts the sociodemographic and clinical characteristics of the study participants.

**Figure 1:**
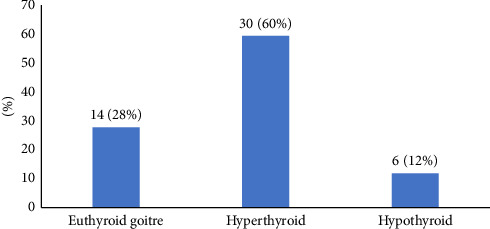
Frequency of thyroid disorders. Euthyroid goitre is an enlargement of the thyroid gland with normal levels of thyroid hormones. Hyperthyroidism is defined by FT4 > 22.0 pmol/L, FT3 > 6.0 pmol/L and TSH < 0.35 µIu/mL [19]. Hypothyroidism is defined by FT3 < 2.9 pmol/L, FT4 < 6.5 pmol/L and TSH > 10 µIu/mL.

The majority of the subjects had clinical and biochemical evidence of hyperthyroidism (60%) followed by those with euthyroid goitre (28%) and those with hypothyroidism (12%) as depicted in Figure 1.

**Figure 2:**
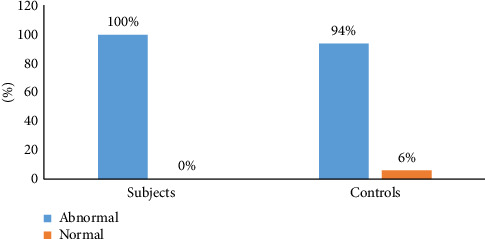
Comparison of selenium levels between subjects and control. Abnormal and normal indicate those with serum selenium below and above the reference range of < 80 μg/L, respectively.

Selenium levels were significantly lower among the cases than in controls (24.9 ± 15.7 μg/L and 59.0 ± 35.9 μg/L, respectively, *p* < 0.001), and the selenium levels were also generally abnormal in the majority of controls as shown in Figure 2. The majority of the controls had selenium levels below the normal reference values.

Table 2 shows the comparison of mean selenium levels in the different clinical categories of thyroid disorders; the mean selenium levels were lowest in subjects with hypothyroidism followed by those with hyperthyroidism while subjects with euthyroid goitre had the highest mean level of selenium.

## 4. Discussion

This cross-sectional study investigated the selenium levels of the different thyroid disorders in a cohort of individuals from Southwest Nigeria. Our findings showed low levels of selenium in the cases compared to controls. This observation could be attributable to general nutritional deficiency in micronutrients in the population that might have remained obscure and unattended.

There were more females presenting with thyroid disease during this study; this is consistent with earlier reports of a higher incidence and prevalence of thyroid disorders in women [24]. This observation is explained by the dimorphism imposed by sex hormones on the immune response which makes women more prone to autoimmune diseases in general and thyroid diseases in particular [25, 26]. The majority of thyroid disorder observed during this study was hyperthyroidism, and this is similar to the report of Okafor and coworkers from the southeast of Nigeria in a hospital-based study, in which, out of the 260 patients studied, 58% had hyperthyroidism secondary to Graves' disease followed by hypothyroidism (39%) [1]. Contrarily, a study from the northern part of Nigeria which looked at the pattern of thyroid disease in Kano found that the majority presented with simple nontoxic goitre, while a retrospective study of patients who presented to a tertiary facility over a 10-year period in Southwest Nigeria also reported that 58.9% presented with simple goitre while 36.6% had toxic goitre and only 1.7% had hypothyroidism [27, 28]. The variability of these findings may be explained by the different sample sizes, different case definitions and different groups studied.

The selenium status in all the cases in this study was below the World Health Organization (WHO) recommended levels. In contrast, however, a recent analysis of subjects from the United States National Health and Nutrition Examination Survey (NHANES) indicated higher serum selenium levels among the population [29]. Much higher serum selenium levels were also reported in a European cohort study to assess the effect of prediagnostic selenium status on the risk of developing breast cancer [30]. The observed values of 25–60 μg/L in this study would be considered as very severely deficient compared with the US and European report stated above. The differences in selenium levels may be attributable to better nutrition available to people in these high-income countries. Several studies of patients with thyroid disorders ranging from endemic goitre to autoimmune thyroid diseases and even thyroid cancers have been linked to selenium deficiency [12]. For instance, myxoedematous endemic cretinism in areas of goitre endemicity in Zaire, East Africa, was associated with low serum selenium and glutathione peroxidase levels; similarly, Thilly et al. demonstrated that endemic goitre was dependent not only on iodine deficiency and thiocyanate overload but also on selenium deficiency [31, 32]. Other European studies are in agreement with this study; Rasmussen and coworkers measured selenium levels before and after iodine fortification program was introduced in Denmark; they thereafter assessed the relationship between selenium levels and the risk for an enlarged thyroid gland as well as development of thyroid nodules. Their study demonstrated that low selenium levels were significantly associated with a larger thyroid volume, higher risk for enlarged thyroid gland and for the development of multiple thyroid nodules in the 1100 women but not in the 792 men who participated [33]. Another study from Prague which measured selenium in the hair, serum and urine of 380 participants in a low selenium region reported an inverse correlation between thyroid hormone levels and selenium [34]. Reports from China indicate increased prevalence of thyroid disease in population-based study in regions with low selenium status; similarly, a prospective 6-year cohort study demonstrated a higher incidence of Hashimoto thyroiditis in the population [35, 36]. The threshold of selenium which would predispose to thyroid disease has however not been clearly elucidated.

The mean serum selenium levels were lower in this study among subjects with hypothyroidism compared with those with hyperthyroidism and euthyroid goitre. Similarly, the study from China which involved patients recruited from an adequate selenium county versus those from a low selenium county concluded that the most prevalent disorders in the low selenium county were overt hypothyroidism, subclinical hypothyroidism and autoimmune thyroiditis [35]. In contrast, however, a prospective comparative register-based population study from Denmark reported that selenium was considerably lower in those with Graves' disease compared with autoimmune hypothyroidism although both conditions demonstrated lower selenium levels than normal controls [37].

Currently, selenium is recommended in the treatment of mild active Graves' orbitopathy of short duration, and several studies have demonstrated the effects of selenium supplementation [12–15] which include reduction in the severity of symptoms, improvements in the quality of life and reduction in markers of autoimmunity which may indicate a possible role for supplementation in Nigerian patients with thyroid disorders. The result of this study demonstrated significant selenium deficiency in all the study subjects.

### 4.1. Limitation and Strength

The relatively small sample size needs to be considered before generalising the outcome. However, this study can serve as a template for further population-based studies to confirm the selenium status of Nigerians with thyroid disease and also to determine the population reference for selenium levels in Nigerians. The study has also provided further evidence that Nigerians are generally deficient in selenium based on current WHO recommended reference range.

## 5. Conclusions

There is significant selenium deficiency in all the subjects with thyroid disease; healthy Nigerians are generally deficient in selenium. Those with hyperthyroidism tended to be more deficient. Selenium supplementation is recommended for patients with thyroid diseases and possibly to fortify salt for the general population as being done currently with iodine.

## Figures and Tables

**Table 1:** Sociodemographic and clinical characteristics of the study participants.

	Subject, *n* (%)	Control, *n* (%)	*χ* ^2^	*p* value
Age group				
≤ 30	10 (20.0)	25 (25.0)	3.170	0.529
31–40	16 (32.0)	36 (36.0)		
41–50	17 (34.0)	31 (31.0)		
51–60	6 (12.0)	8 (8.0)		
> 60	1 (2.0)	0 (0.0)		
Gender				
Male	10 (20.0)	28 (28.0)	1.128	0.288
Female	40 (80.0)	72 (72.0)		
Duration of stay at current address in years				
≤ 5	6 (12.0)	44 (44.0)	21.202	0.003
6–10	17 (34.0)	28 (28.0)		
11–15	13 (26.0)	8 (8.0)		
16–20	5 (10.0)	9 (9.0)		
21–25	4 (8.0)	5 (5.0)		
26–30	3 (6.0)	5 (5.0)		
31–35	1 (2.0)	0 (0.0)		
36–40	1 (2.0)	1 (1.0)		
Level of education				
No formal	5 (10.0)	2 (2.0)	8.651	0.034
Primary	1 (2.0)	0 (0.0)		
Secondary	13 (26.0)	19 (19.0)		
Tertiary	31 (62.0)	79 (79.0)		

*Note:* Level of education indicates those who commenced or completed each category. Primary—6 years, secondary—6 years and tertiary—at least 4 years.

**Table 2:** Comparison of mean selenium levels in various types of thyroid disorders.

	Euthyroid goitre, mean ± SD	Hyperthyroid, mean ± SD	Hypothyroid, mean ± SD	*F*	*p* value
Selenium	27.67 ± 14.69	24.22 ± 17.19	22.32 ± 11.25	0.314	0.732

*Note:* Selenium (μg/L).

## Data Availability

The data that support the findings of this study are available from the corresponding author upon reasonable request.
